# From Sewage to Salvage: Complete Characterization of Arefeen1 Phage Against MDR *Pseudomonas aeruginosa*

**DOI:** 10.1155/ijm/8509161

**Published:** 2026-04-24

**Authors:** Arefeen Haider, Tahsin Khan, Naimul Islam Faysal, Sezanur Rahman, Jannat Akther, Shovan Basak Moon, Nure Sharaf Nower Samia, Shakhinur Islam Mondal, Anowara Begum, Sudhangshu Kumar Biswas, Mustafizur Rahman, Mohammad Jubair

**Affiliations:** ^1^ Genome Centre, Infectious Diseases Division, International Centre for Diarrhoeal Disease Research Bangladesh, Mohakhali, Dhaka, 1212, Bangladesh; ^2^ Virology Laboratory, Infectious Diseases Division, International Centre for Diarrhoeal Disease Research Bangladesh, Mohakhali, Dhaka, 1212, Bangladesh; ^3^ Department of Genetic Engineering and Biotechnology, Shahjalal University of Science Technology, Sylhet, Bangladesh; ^4^ Department of Microbiology, University of Dhaka, Dhaka, Bangladesh, du.ac.bd; ^5^ Bacteriophage Biology and Genomics Lab, Department of Biotechnology and Genetic Engineering, Islamic University, Kushtia, Bangladesh, iu.ac.bd

**Keywords:** lytic bacteriophage, MDR *Pseudomonas aeruginosa*, phage therapy, therapeutic phage characterization, wastewater bioprospecting

## Abstract

The escalating global threat of multidrug‐resistant (MDR) *Pseudomonas aeruginosa*, designated by the WHO as a critical‐priority pathogen, necessitates urgent development of alternative therapeutics. We present *Pseudomonas* phage Arefeen1, isolated from Bangladeshi wastewater, as a clinically translatable candidate with therapeutic potential. Comprehensive genomic characterization revealed a 46,021 bp strictly lytic genome (52.68% GC content) belonging to the Caudoviricetes class, completely lacking virulence factors or antibiotic resistance genes as confirmed by VFDB and ResFinder screening—a crucial safety profile for clinical application. Phenotypically, Arefeen1 demonstrated efficient in vitro lytic activity, exhibiting rapid replication kinetics (25‐min latent period), high burst size (150 ± 12 PFU/cell), and robust production yields (≥ 10^9^ PFU/mL post‐PEG precipitation). Host range analysis showed 85.7% efficacy against a panel of clinically relevant MDR strains, including respiratory (PA_CU_1) and wound (MO_4642.012.002) isolates. Comparative genomics with 840 *P. aeruginosa* phages identified six unique genes encoding membrane‐interaction proteins (CDS_0052/0058/0061) and a specialized tRNA complement, suggesting evolutionary adaptations for enhanced host range and translational efficiency. Its isolation from Bangladesh’s unique microbial ecosystem provides a geographically optimized resource for LMICs disproportionately affected by antimicrobial resistance. These findings, combined with its clinical strain coverage, position Arefeen1 as a potential candidate for preclinical development and phage therapy implementation against this formidable pathogen.

## 1. Introduction

The increasing prevalence of multidrug‐resistant (MDR) bacterial pathogens has necessitated the exploration of alternative antimicrobial strategies, with bacteriophage (phage) therapy emerging as a promising solution. Phages, viruses that specifically infect and lyse bacteria, are the most abundant biological entities on Earth and play a crucial role in regulating microbial populations and driving bacterial evolution [1, 2]. Among clinically relevant pathogens, *Pseudomonas aeruginosa* poses a significant threat due to its intrinsic resistance to antibiotics and ability to acquire additional resistance mechanisms [3]. Recognized by the World Health Organization (WHO) as a critical‐priority MDR pathogen, *P. aeruginosa* is a leading cause of nosocomial infections, chronic respiratory diseases, and sepsis, particularly in immunocompromised individuals [4]. The dwindling efficacy of conventional antibiotics against such strains has revitalized interest in phage therapy, which offers host‐specific lytic activity without the broad‐spectrum disruption of the microbiome [5]. This interest is supported by a growing body of contemporary research isolating and characterizing *Pseudomonas* phages for therapeutic applications, including studies on synergistic phage‐antibiotic combinations [6], the discovery of broad‐spectrum lytic phages with antibiofilm efficacy [7, 8], and the development of advanced delivery systems like phage‐lysin hydrogels [9].

Environmental reservoirs, particularly wastewater systems, serve as hotspots for phage diversity due to the high density of bacteria and dynamic phage–host interactions [10]. These environments provide ideal conditions for phage proliferation, as the constant influx of bacterial hosts and nutrient‐rich settings facilitate rapid coevolution between phages and their targets [11]. Wastewater from hospitals and urban areas is especially rich in clinically relevant bacterial strains, including MDR *P. aeruginosa*, making it a strategic source for isolating phages with therapeutic potential [12].

Environmental surveillance in regions with high AMR burden, such as Bangladesh, is a strategic source for discovering novel therapeutic phages. While studies in Dhaka have identified potent *Pseudomonas* phages active against MDR strains, the systematic characterization of locally isolated phages remains limited [13, 14]. Isolating and characterizing phages from these distinct ecological settings addresses a key scientific gap by expanding the global repertoire of well‐defined phages and revealing unique adaptations to local bacterial populations [15].

In this study, we isolated and characterized *Pseudomonas* phage Arefeen1 from wastewater in Dhaka, Bangladesh, with the aim of evaluating its genomic features, evolutionary relationships, and therapeutic potential. Through whole‐genome sequencing, comparative genomics, and host range assays, we assessed its lytic efficacy against clinical *P. aeruginosa* strains and identified unique genomic elements that may contribute to host adaptation. Our findings expand the repository of phages with therapeutic potential and provide insights into the genetic diversity of *P. aeruginosa*‐infecting phages. By bridging genomic analysis with functional validation, this work underscores the importance of phage discovery in combating the global AMR crisis.

## 2. Methods

### 2.1. Sample Collection and Phage Isolation

Wastewater samples were collected aseptically from sewage sites in Dhaka, Bangladesh, in 2024 using sterile 500 mL polypropylene containers (Thermo Fisher Scientific). Samples were transported on ice to the laboratory and processed within 3 h of collection to maintain phage viability. Phage isolation was performed using the enrichment protocol described by Son et al. [16] with modifications. Briefly, 10 mL of each sample was mixed with 5 mL of log‐phase *P. aeruginosa* (host strain obtained from the icddr,b Genome Centre Biobank) in Lysogeny Broth (LB). After 24 h of incubation at 37°C with shaking (180 rpm), the mixture was centrifuged (10,000 × *g*, 10 min), and the supernatant was filtered through a 0.22 μm polyethersulfone membrane (Millipore) to remove bacterial debris. Phage presence was confirmed by plaque formation using the double‐layer agar method [17], with LB agar (1.5% *w*/*v*) as the base layer and LB top agar (0.7% *w*/*v*) containing 100 μL of the filtered supernatant and 200 μL of host culture. Plates were incubated overnight at 37°C, and well‐isolated plaques were picked for further purification.

### 2.2. One‐Step Growth Curve

The one‐step growth curve was performed as previously described [9]. Briefly, the host bacterium *P. aeruginosa* PAO1 was grown to mid‐exponential phase (OD600 ∼0.3) in LB broth at 37°C. The culture was infected with phage Arefeen1 at a multiplicity of infection (MOI) of 0.1 and allowed to adsorb for 5 min at 37°C without shaking. The mixture was then centrifuged at 8000 × *g* for 2 min to remove unadsorbed phages, and the pellet was resuspended in fresh prewarmed LB. This time point was designated *t* = 0. Resuspended infected cells were diluted 1:1000 into prewarmed LB and incubated at 37°C with shaking. Samples (100 μL) were taken at 5‐min intervals for 60 min, immediately diluted in SM buffer, and plated using the double‐layer agar method [17, 18] to determine the phage titer (PFU/mL). The latent period was defined as the time from the start of the infection until the first detectable increase in progeny phage titer. The burst size was calculated as the ratio of the final phage titer after lysis to the initial titer of infected cells. All experiments were performed in biological triplicate.

### 2.3. Host Range and Efficiency of Plating (EOP) Assay

The host range of phage Arefeen1 was determined against a panel of seven clinically isolated, MDR *P. aeruginosa* strains (Table 1) using a spot test and a quantitative EOP assay. For the spot test, 5 μL of a high‐titer phage lysate (≥ 10^8^ PFU/mL) was spotted onto lawns of each bacterial strain prepared in LB top agar. Assays were performed in triplicate on separate occasions (*n* = 3 biological replicates). Plates were examined for zones of clearance after overnight incubation at 37°C. For the EOP assay, phage lysates were serially diluted and plated on each bacterial strain using the double‐layer agar method. The EOP was calculated as the phage titer on the test strain divided by the phage titer on the primary host strain (*P. aeruginosa* PAO1). EOP values were categorized as high (EOP ≥ 0.5), moderate (0.1 ≤ EOP < 0.5), low (0.001 ≤ EOP ≤ 0.1), or inactive (EOP ≤ 0.001) [19].

**TABLE 1:** Host range and replication efficiency of bacteriophage Arefeen1.

Host strain	Designation	Spot test result	EOP value	EOP classification
*P. aeruginosa*	PA_CU_1	Positive	0.7	High
*P. aeruginosa*	PA_CU_2	Positive	0.4	Moderate
*P. aeruginosa*	PA_CU_3	Positive	0.9	High
*P. aeruginosa*	PA_CU_5	Positive	0.08	Low
*P. aeruginosa*	DU_16	Positive	0.2	Moderate
*P. aeruginosa*	Pse	Negative	< 0.001	Inactive
*P. aeruginosa*	MO_4642.012.002	Positive	0.6	High

### 2.4. DNA Extraction and Whole Genome Sequencing

A single plaque was excised and eluted in SM buffer (50 mM Tris‐HCl, 100 mM NaCl, 8 mM MgSO_4_, pH 7.5), followed by plaque purification to ensure clonal isolation. High‐titer phage lysates were prepared by infecting log‐phase *P. aeruginosa* at an MOI of 0.01 and incubating until complete lysis (6–8 h). Phage particles were concentrated via polyethylene glycol (PEG8000) precipitation [20] and resuspended in SM buffer. Prior to DNA extraction, the purified lysate was treated with DNase I (1 μg/mL, 37°C for 1 h) to eliminate residual bacterial genomic DNA. Genomic DNA was extracted using the DNeasy Blood & Tissue Kit (Qiagen, Germany) following the manufacturer’s protocol, with an additional RNase A treatment. DNA quality was assessed by Nanodrop (A_260_/A_280_ ratio > 1.8) and Qubit fluorometry (Thermo Fisher Scientific). Libraries were prepared with the Illumina DNA Prep Kit (Illumina) and sequenced on an Illumina MiSeq platform (2 × 250 bp paired‐end reads; 500‐cycle kit).

### 2.5. Quality Control, Genome Assembly, and Annotation

Raw reads were quality‐checked using FastQC v0.11.9 [21], followed by trimming with Trimmomatic v0.39 [22] (parameters: SLIDINGWINDOW:4:20 HEADCROP:20 CROP:220 MINLEN:150). The filtered reads were assembled de novo with SPAdes v3.15.5 [23] using—careful—only‐assembler and k‐mer 21, 33, 55, 77, 87, 99, 111, 119, and 127. Depth coverage was calculated with BBmap v37.62 [24], and the contig with the highest depth was selected for further analysis. Assembly quality was assessed using QUAST v5.2.0 [25], and genome completeness was verified with CheckV v1.0.1 [26]. Genome annotation was performed using Pharokka v1.7.3 [27]. Subsequently, genome reorientation was conducted using Circlator [28] with the terminase small subunit as the starting point, followed by a second round of annotation to ensure consistency.

### 2.6. Virulence and Lifestyle Prediction

The phage lifestyle (lytic vs. temperate) was predicted using BACPHLIP v0.9.6 [29] and PhaTYP [30], with confidence thresholds of > 90%. Protein domains were identified using HMMER v2.41.2 [31] and InterProScan v5.64‐96.0 [32], while screening for antimicrobial resistance genes and virulence factors was conducted using the ResFinder [33] and VFDB [34] databases within Abricate v1.0.1 [35].

### 2.7. Pan‐Genome, Phylogenetic, and Comparative Genome Analysis

A total of 984 *P. aeruginosa* phage genomes were selected from the INPHARED database [36]. After excluding 144 partial genomes, 840 complete genomes were retained. Virulent phages (*n* = 678) were identified using BACPHLIP and PhaTYP. Proteomic trees were generated with ViPTree v1.1.3 [37], narrowing down a clade of 58 phages closely related to Arefeen1. Intergenomic nucleotide distances were calculated using VIRIDIC [38] with default parameters (genus threshold: 70%, species threshold: 95%), resulting in a final clade of 48 closely related phages. Genomes were then reoriented using Circlator, with the terminase small subunit as a reference, to standardize genome orientation for comparative analysis. All genomes were re‐annotated in Pharokka to ensure consistency.

A pan‐genome analysis was performed on 48 *P. aeruginosa* phage genomes. After annotation, the GFF files generated by Pharokka were used as input for creating the pan‐genome with Roary v3.13.0 software [39]. Annotated genomes from Pharokka were processed in Roary with the following parameters: ‐e ‐n ‐v ‐s ‐i 60 ‐cd 100. Genes present in 99%–100% of the genomes were classified as core genes, 95%–99% as soft‐core, 15%–95% as shell, and less than 15% as cloud genes. The distribution of core and accessory genomes was visualized using Phandango [40], with additional comparative visualizations generated in gggenomes [41].

## 3. Results

### 3.1. Isolation of *Pseudomonas* Phage Arefeen1

We isolated *Pseudomonas* phage Arefeen1 from wastewater in Dhaka, Bangladesh, using *P. aeruginosa* PAO1 as the host strain. Plaque purification yielded uniform lytic zones averaging 2–3 mm in diameter (Figure 1(a)), consistent with typical podovirus morphology.

FIGURE 1(a) Plaque morphology and lytic activity of phage Arefeen1. (A, B) Plaque formation on *P. auruginosa* double‐layer agar plates at time intervals of 30 and 80 min. Clear, circular plaques (0.5–1.5 mm in diameter) were observed after 16–18‐h incubation at 37°C; (C) Spot test showing a distinct lysis zone, confirming the virulent activity of phage Arefeen1 against its host strain. (b) One‐step growth curve of bacteriophage Arefeen1 infecting *P. aeruginosa*.

**Figure:**
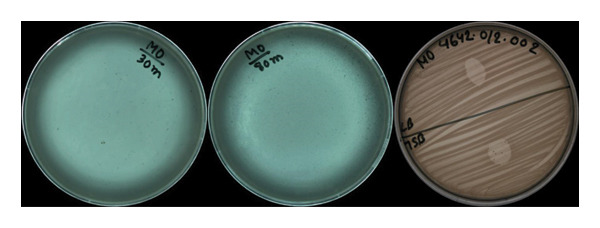
(a)

**Figure:**
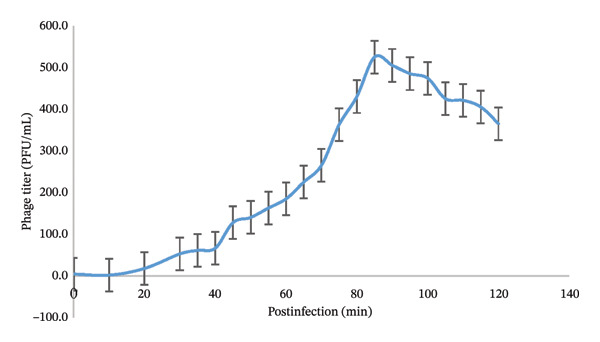
(b)

### 3.2. One‐Step Growth Curve

The one‐step growth curve of bacteriophage Arefeen1 infecting *P. aeruginosa* revealed a 25‐min latent period. A subsequent rise phase culminated in a peak titer at ∼85 min post‐infection. Based on this increase, the average burst size was estimated to be 150 ± 12 PFU per infected cell (mean ± SD of three independent biological replicates) (Figure 1(b)), indicating efficient replication.

### 3.3. Phenotypic Characterization

The host range and replication kinetics of Arefeen1 were systematically evaluated to assess its therapeutic potential. Direct spot testing against seven clinical *P. aeruginosa* isolates revealed lytic activity, with clear plaque formation observed in six strains (85.7% efficacy), including respiratory (PA_CU_1) and wound (MO_4642.012.002) isolates. The single resistant strain (Pse, bloodstream isolate) may possess phage defense mechanisms such as receptor modification or CRISPR‐Cas systems (Table 1).

Three strains (PA_CU_1, PA_CU_3, and MO_4642.012.002) supported high‐efficiency replication (EOP 0.6–0.9). Two strains (PA_CU_2 and DU_16) showed moderate efficiency (EOP 0.2–0.4), while strain PA_CU_5 supported low‐efficiency plating (EOP 0.08). A single bloodstream isolate (Pse) was completely resistant, showing no lytic activity and an EOP below 0.001 (Table 1).

### 3.4. Genome Annotation, Taxonomy, and Functional Analysis

Whole‐genome sequencing revealed a 46,021 bp double‐stranded DNA genome with 52.7% GC content, achieving high‐quality assembly (353 × coverage, CheckV completeness: 100%). Taxonomic analysis placed Arefeen1 within the genus *Bruynoghevirus* (class Caudoviricetes), showing 95%–98% nucleotide identity to reference phages (Table 2). Lifestyle prediction tools BACPHLIP (98.7% confidence) and PhaTYP (99.99% confidence) confirmed its obligately lytic nature, with no detectable integrase or antibiotic resistance genes—key attributes for therapeutic applications.

**TABLE 2:** Genomic features and taxonomic classification of Arefeen1 compared to related phages.

Parameter	Arefeen1 OR472559	Phage_Pa222	Phage_SaPL	Phage_vB_PaeP_PaCe
Genome size (bp)	46,021	—	—	—
Coverage	353 ×	—	—	—
GC content (%)	52.7	—	—	—
CheckV completeness (%)	100	—	—	—
BacPHLIP (% virulent)	98.7	—	—	—
PhaTYP (% virulent)	99.99	—	—	—
Query coverage (%)	—	98	96	96
Percent identity (%)	—	97.4	98.5	95.5

**FIGURE 2:**
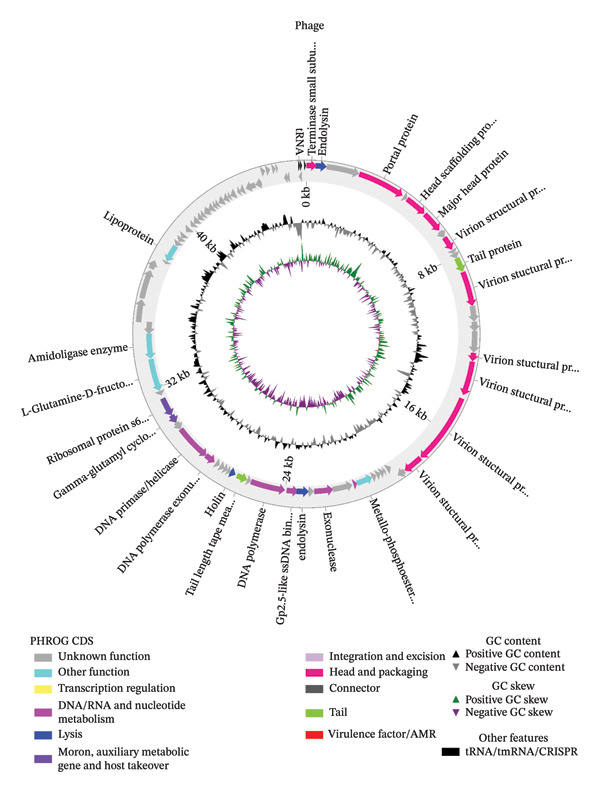
Genome annotation of Arefeen1 showing functional modules. The 46,021 bp genome contains 86 ORFs (82 protein‐coding, 4 tRNA), covering 96.26% of the sequence. Key features include structural proteins (12 ORFs), replication systems (6 ORFs), lytic enzymes (3 ORFs), and metabolic genes (4 ORFs), with no virulence or antimicrobial resistance factors detected.

The annotated genome of Arefeen1 contained 86 open reading frames (ORFs) spanning 96.26% of the 46,021 bp sequence, with protein‐coding sequences averaging 515.1 bp in length (Figure 2). Among these, we identified 82 protein‐coding genes and 4 tRNA genes (including one pseudogene) shown in Supplementary Figure 1, distributed asymmetrically across strands (32 ORFs on the positive strand vs. 54 on the negative strand). Functional classification revealed three key modules: (1) structural components (12 ORFs), including 10 head/packaging proteins and 2 tail‐associated proteins; (2) replication/regulation systems (6 ORFs); and (3) a lytic module featuring 2 endolysins and 1 holin. Additionally, four metabolic enzymes were annotated—an amidoligase, a metallo‐phosphoesterase, L‐glutamine‐D‐fructose‐6‐phosphate aminotransferase, and lipoprotein—suggesting potential roles in host metabolic redirection during infection. Crucially, comprehensive screening against the VFDB and ResFinder databases confirmed the absence of both antimicrobial resistance genes and known virulence factors, reinforcing the phage’s therapeutic safety profile.

To definitively establish the taxonomic position of Arefeen1, we generated a proteomic tree with ViPTree [37] and placed Arefeen1 within a well‐defined clade corresponding to the genus *Bruynoghevirus* [42] (Figure 3(a)). Consistent with this, we calculated pairwise intergenomic similarities using VIRIDIC [38] against all complete *P. aeruginosa* phage genomes (Figure 3(b)). Arefeen1 shared > 96% average nucleotide identity with phages Pa222, HMGUpa2, and PA_CQ9, exceeding the 95% threshold recommended for species demarcation [38]. Therefore, we classify Arefeen1 as a new strain within the established species *Pseudomonas* virus Pa222, genus *Bruynoghevirus*.

FIGURE 3Comparative genomic analysis of phage Arefeen1. (a) Proteomic phylogenetic tree aligned with genome similarity, showing taxonomic clustering. (b) VIRIDIC heatmap of intergenomic similarity among Arefeen1 and related *P. aeruginosa* phages, with identity percentages, genome lengths, and aligned fractions.

**Figure:**
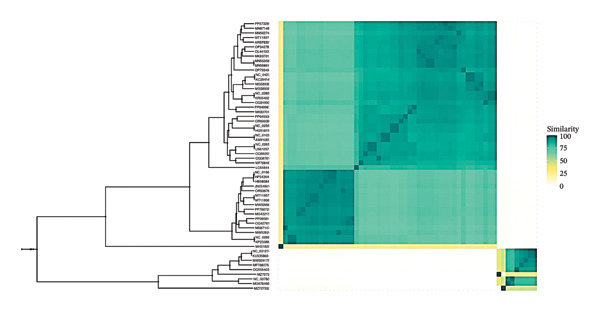
(a)

**Figure:**
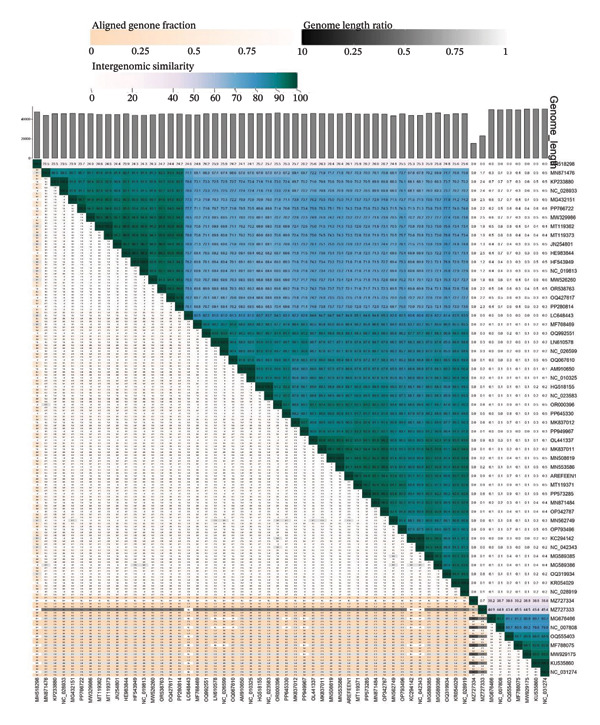
(b)

### 3.5. Comparative Genomic Analysis

**FIGURE 4:**
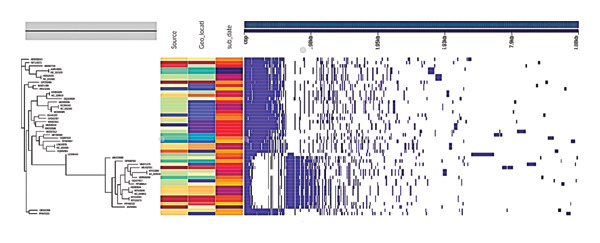
The pan‐genome analysis of 48 related phages, constructed based on intergenomic similarity, uses hierarchical clustering (left panel) and presence–absence matrices (center/right) with annotation for source, location, and date to illustrate a conserved core genome and a diverse accessory genome contributing to functional specialization.

Pan‐genome analysis of 48 closely related *P. aeruginosa* phages, selected from 840 complete genomes in the INPHARED database, revealed distinct patterns of gene conservation and variability (Figure 4). The analysis identified a small core genome of just 5 genes (99%–100% prevalence across all phages), including the essential terminase large subunit (Group_161) involved in DNA packaging. A larger set of 152 shell genes (15%–95% prevalence) and 335 cloud genes (< 15% prevalence) demonstrated substantial accessory genome variability, suggesting evolutionary adaptation to specific ecological niches. Notably, six genes were found to be completely unique to Arefeen1, potentially encoding phage‐specific adaptations that contribute to its distinct biological properties. This genomic architecture—featuring a highly conserved core alongside variable accessory genes—mirrors patterns observed in other virulent phages and supports the evolutionary flexibility of *P. aeruginosa* phages in responding to diverse host environments.

### 3.6. Functional Characterization of Core Genes

Detailed analysis of the conserved core genes revealed several functionally important proteins. The terminase large subunit (Group_161, 482 aa) contained two critical domains: a Terminase_6C domain involved in DNA binding and cleavage during genome packaging and a P‐loop NTPase domain responsible for ATP hydrolysis to power DNA translocation. The portal protein (Group_112, 706 aa) featured predicted coiled‐coil motifs indicative of oligomerization capability, essential for forming the capsid portal complex that facilitates DNA entry and exit. Two small hypothetical proteins (Group_113/114, 63–82 aa) were particularly interesting, as they contained identifiable signal peptides, strongly suggesting they function in secretion or membrane interactions. While Group_113/114’s exact roles remain to be determined, their conserved presence across related phages implies important, though potentially nonessential, functions in the phage life cycle.

### 3.7. Unique Genes and Potential Adaptations

The genome of Arefeen1 contains six unique genes (AREFEEN1_CDS_0052‐0079) that lack homologs in related phages, all encoding hypothetical proteins of currently unknown function. Structural bioinformatics analyses provide important clues about their potential roles: three genes (CDS_0052, 0058, and 0061) contain predicted transmembrane domains, suggesting involvement in membrane interactions during host recognition or viral entry. CDS_0079 displays intrinsically disordered regions that may facilitate structural flexibility and host adaptation, while CDS_0058 shows sequence features consistent with potential involvement in phage replication processes. Although the precise functions of these genes remain to be experimentally validated, their exclusive presence in Arefeen1 indicates they may confer unique adaptive advantages, possibly contributing to host range specificity or environmental fitness. The identification of these phage‐specific genes highlights the genetic diversity among *Pseudomonas* phages and underscores the need for further functional characterization of these potentially important genomic elements.

## 4. Discussion

The increasing prevalence of MDR *P*. *aeruginosa* necessitates the continuous expansion of the global phage repertoire as a potential therapeutic resource [43, 44]. In this study, we isolated and characterized *Pseudomonas* phage Arefeen1 from a Bangladeshi wastewater source. Its strictly lytic nature, absence of virulence or resistance genes, and efficient in vitro activity against a panel of clinical MDR strains support its further investigation as a potential biocontrol agent.

The microbiological characteristics of Arefeen1 are consistent with those of other virulent *P. aeruginosa* podoviruses used in therapy research. Its latent period of 25 min and burst size of ∼150 PFU/cell are efficient and comparable to other therapeutic candidates, such as phage PEV20 (latent period ∼20 min) [45], indicating robust replicative potential. Notably, Arefeen1 lysed 6 out of 7 (85.7%) clinically derived MDR strains in our panel, including respiratory and wound isolates. This host range is similar to that reported for phages like JG004 and PAK_P1, which also show activity against a broad spectrum of clinical isolates ([18]). The single resistant strain (Pse) underscores a common challenge in phage therapy—pre‐existing or rapidly inducible bacterial resistance—which likely arises from bacterial defense mechanisms such as receptor modification, restriction‐modification systems, or CRISPR‐Cas adaptive immunity [46]. While not directly tested in this initial characterization, the presence of such a resistant strain within a small panel highlights the importance of developing phage cocktails [47], rather than monotherapies, to mitigate resistance emergence.

We acknowledge that the planktonic culture conditions used in this initial characterization, while standard for phage isolation and basic kinetic studies, do not recapitulate the complex biofilm mode of growth central to many recalcitrant *P. aeruginosa* infections, such as those in cystic fibrosis airways or chronic wounds [48]. The efficacy of phage therapy can be significantly different in biofilm environments due to factors like reduced phage penetration, altered bacterial metabolism, and the presence of persister cells. Therefore, the promising in vitro activity reported here does not directly predict efficacy in these more clinically relevant settings. Future work must include structured biofilm assays to evaluate Arefeen1’s biofilm penetration and eradication capabilities, both alone and in combination with other phages or antibiotics known to disrupt biofilm matrices.

Genomically, Arefeen1 is a member of the *Bruynoghevirus* genus, sharing high average nucleotide identity (> 96%) with phages like Pa222 and PA_CQ9. This taxonomic assignment places it within a group known to infect *P. aeruginosa* [8]. Its genome encodes several features commonly associated with virulent phages, including a suite of tRNAs, which are not innovations but recognized adaptations that may enhance translational efficiency in a GC‐rich host [49]. Pan‐genome analysis identified six clade‐specific ORFs in Arefeen1, three of which (CDS_0052, 0058, and 0061) encode predicted transmembrane domains—a known feature of phage host‐interaction proteins—suggesting recent acquisition or divergence and a potential role in fine‐tuning host range, though their definitive function requires validation. This unique set resides within a genome where approximately 64% of ORFs are hypothetical proteins, reflecting the common, vast unexplored diversity of phage genomics. The conserved core genome, comprising efficient structural and replication modules, underpins the lytic kinetics, while conserved small proteins with predicted signal peptides indicate a potentially novel, nonstructural mechanism in this phage group’s infection cycle, meriting further study [10, 48].

The primary significance of this work lies in its geographical and clinical context. Bangladesh faces a severe burden of AMR [14]. Isolating and characterizing phages from local environmental reservoirs, such as the Dhaka sewage system, is a strategic approach to developing regionally relevant therapeutic resources. Phages co‐evolve with local bacterial populations, making indigenous isolates potentially more effective against circulating clinical strains [21]. While the clinical application of phages against *P. aeruginosa* is advancing, as demonstrated in compassionate‐use cases for pneumonia and burn wound infections [5, 8], these successes rely on well‐characterized phage banks. Arefeen1 represents a new, genomically defined addition to such banks, specifically sourced from and for a high‐AMR burden region.

We acknowledge the limitations of this initial characterization. Transmission electron microscopy (TEM) was not performed for phage morphological analysis. The host range, while encouraging, was tested against 6 of 7 tested MDR clinical isolates, which is a limited number of strains. The in vitro lytic activity does not guarantee efficacy in complex in vivo or biofilm environments [48]. Furthermore, the functional predictions for core and unique genes remain to be tested experimentally.

In conclusion, *Pseudomonas* phage Arefeen1 is a genomically and phenotypically characterized, strictly lytic virus with activity against clinically relevant MDR strains in Bangladesh. Its value is not in unprecedented novelty but in its contribution as a validated, regionally sourced candidate for inclusion in future phage cocktail formulations. Future work should focus on combinatorial synergy testing with other phages or antibiotics, in vivo efficacy models, structured biofilm assays, and functional genomics to elucidate the roles of its uncharacterized genes. This study underscores the importance of systematic local phage bioprospecting as a component of the multifaceted response to the AMR crisis.

## 5. Conclusion

The study comprehensively characterizes *Pseudomonas* phage Arefeen1, isolated from Bangladeshi wastewater, as a therapeutic candidate against MDR *P. aeruginosa*. Arefeen1 exhibits a lytic lifecycle, a 46,021 bp genome devoid of virulence or antibiotic resistance genes, and robust lytic activity against 85.7% of tested clinical strains. Its rapid replication kinetics (25‐min latent period, burst size of 150 ± 12 PFU/cell) and genomic adaptations, including six membrane‐interaction genes, highlight its therapeutic potential. Comparative genomics revealed a small core genome but significant accessory gene diversity, underscoring evolutionary adaptability. These findings position Arefeen1 as a candidate for preclinical development, particularly in regions disproportionately affected by antimicrobial resistance.

## Author Contributions

Arefeen Haider, Sezanur Rahman, and Mohammad Jubair developed the protocol and methodology, conceived and coordinated the study, and reviewed the manuscript. Mohammad Jubair, Arefeen Haider, Tahsin Khan, and Naimul Islam Faysal interpreted laboratory data, cleaned and finalized the dataset, performed the descriptive analyses, and prepared the first draft of the manuscript. Tahsin Khan, Naimul Islam Faysal, and Nure Sharaf Nower Samia analyzed all genomic data. Arefeen Haider, Jannat Akther, and Shovan Basak Moon were involved in the laboratory work and analysis of laboratory data and provided intellectual input to the manuscript. Mustafizur Rahman, Shakhinur Islam Mondal, Anowara Begum, and Sudhangshu Kumar Biswas critically reviewed the manuscript and provided intellectual input. All authors had full access to all the data in the study and accepted the responsibility for the integrity of the data, accuracy of the data analysis, and publication.

## Funding

This research did not receive any specific funding. However, icddr,b relies on core unrestricted support from the Governments of Bangladesh and Canada to sustain its hospital operations and broader institutional functions.

## Disclosure

All authors reviewed subsequent drafts of the manuscript and approved the final version of the manuscript.

## Ethics Statement

This environmental study involved sewage sample collection and analysis, with no human or animal subjects. Ethical approval was not required. Sampling followed local regulations, and permissions were obtained from landowners and authorities.

## Conflicts of Interest

The authors declare no conflicts of interest.

## Supporting Information

Supporting Figure 1. Genome annotation map of phage Arefeen1, indicating the locations of 3 tRNA genes and 1 pseudogene.

## Supporting information


**Supporting Information** Additional supporting information can be found online in the Supporting Information section.

## Data Availability

The accession number of *Pseudomonas* phage Arefeen1 is OR472559 under bioproject PRJNA972335.

## References

[1] Clokie M. R. J. , Millard A. D. , Letarov A. V. , and Heaphy S. , Phages in Nature, Bacteriophage. (2011) 1, no. 1, 31–45, 10.4161/bact.1.1.14942. 21687533 PMC3109452

[2] Suttle C. A. , Marine Viruses: Major Players in Global Ecosystems, Nature Reviews Microbiology. (2007) 5, no. 10, 801–812, 10.1038/nrmicro1750, 2-s2.0-34548792911. 17853907

[3] Breidenstein E. B. M. , de la Fuente-Núñez C. , and Hancock R. E. W. , *Pseudomonas Aeruginosa*: All Roads Lead to Resistance, Trends in Microbiology. (2011) 19, no. 8, 419–426, 10.1016/j.tim.2011.04.005, 2-s2.0-79960923273. 21664819

[4] World Health Organization , Global Priority List of antibiotic-resistant Bacteria, 2017, WHO, Geneva.

[5] Schooley R. T. , Biswas B. , Gill J. J. et al., Personalized Bacteriophage Therapy for Disseminated Resistant *Acinetobacter baumannii* , Antimicrobial Agents and Chemotherapy. (2017) 61, no. 10, e00954–17. 28807909 10.1128/AAC.00954-17 PMC5610518

[6] Holger D. J. , El Ghali A. , Bhutani N et al., Phage-Antibiotic Combinations Against MDR *Pseudomonas aeruginosa* in Static and Dynamic Biofilm Models, Antimicrobial Agents and Chemotherapy. (2023) 67, no. 11, 10.1128/aac.00578-23. PMC10648846 37855639

[7] Shi Z. , Hong X. , Li Z et al., Broad-Spectrum Lytic Phage Pae01 Against *P. aeruginosa* , Frontiers in Microbiology. (2024) 15, 10.3389/fmicb.2024.1386830. PMC11292732 39091310

[8] Kovacs C. J. , Rapp E. M. , Rankin W. R et al., Phage Combinations Enhance Activity Against MDR *Pseudomonas aeruginosa* , Viruses. (2024) 16, no. 7, 10.3390/v16071000. PMC11281517 39066163

[9] Hyman P. and Abedon S. T. , Practical Methods for Determining Phage Growth Parameters, Methods in Molecular Biology, 2009, 501, Humana Press, New York, 175–202, 10.1007/978-1-60327-164-6_18. 19066822

[10] Dion M. B. , Oechslin F. , and Moineau S. , Phage Diversity, Genomics and Phylogeny, Nature Reviews Microbiology. (2020) 18, no. 3, 125–138, 10.1038/s41579-019-0311-5. 32015529

[11] Shapiro O. H. , Kushmaro A. , and Brenner A. , Phage-Host Interactions in Natural and Engineered Systems, Current Opinion in Biotechnology. (2010) 21, no. 4, 382–387.

[12] Jassim S. A. A. , Limoges R. G. , and El-Cheikh H. , Bacteriophages in Sewage: Abundance, Roles, Applications, FEMS Microbiology Ecology. (2016) 92.

[13] Zaman S. B. , Chowdhury T. , Hossain M. N. , Rahman M. , and Moniruzzaman M. , Lytic *Pseudomonas* Phages from Dhaka Wastewater, Journal of Genetic Engineering and Biotechnology. (2022) 20.

[14] Islam M. S. , Zhou Y. , Liang L. , Nime I. , and Liu L. , Application of Phage Therapy in the Era of Antimicrobial Resistance, Frontiers in Microbiology. (2020) 11.

[15] Silveira C. B. and Rohwer F. L. , Piggyback-The-Winner in host-associated Communities, npj Biofilms Microbiomes. (2016) 2, no. 1, 10.1038/npjbiofilms.2016.10, 2-s2.0-85021813111. PMC5515262 28721247

[16] Son J. S. , Lee S. J. , Jun S. Y et al., Antibacterial Activity of Phage SAP-2, Applied Microbiology and Biotechnology. (2010) 86, no. 5, 1439–1449, 10.1007/s00253-009-2386-9, 2-s2.0-77952890163. 20013118

[17] Adams M. H. , Bacteriophages, 1959, Interscience Publishers, New York.

[18] Kropinski A. M. , Mazzocco A. , Waddell T. E. , Lingohr E. , and Johnson R. P. , Enumeration of Bacteriophages by Double Agar Overlay Plaque Assay, Methods in Molecular Biology. (2009) 501, 69–76. 19066811 10.1007/978-1-60327-164-6_7

[19] Khan Mirzaei M. and Nilsson A. S. , Isolation of Phages for Phage Therapy: Spot Test Vs EOP Determination, PLoS One. (2015) 10, no. 3, 10.1371/journal.pone.0118557, 2-s2.0-84924401895. PMC4356574 25761060

[20] Yamamoto K. R. , Alberts B. M. , Benzinger R. , Lawhorne L. , and Treiber G. , Rapid Bacteriophage Sedimentation Using PEG, Virology. (1970) 40, no. 3, 734–744, 10.1016/0042-6822(70)90218-7, 2-s2.0-0014748362. 4908735

[21] Andrews S. F.Q. C. , A Quality Control Tool for High Throughput Sequence Data, Babraham Bioinformatics. (2010) .

[22] Bolger A. M. , Lohse M. , and Usadel B. , Trimmomatic: A Flexible Trimmer for Illumina Sequence Data, Bioinformatics. (2014) 30, no. 15, 2114–2120, 10.1093/bioinformatics/btu170, 2-s2.0-84905049901. 24695404 PMC4103590

[23] Bankevich A. , Nurk S. , Antipov D et al., Spades: A New Genome Assembly Algorithm and its Applications to single-cell Sequencing, Journal of Computational Biology. (2012) 19, no. 5, 455–477, 10.1089/cmb.2012.0021, 2-s2.0-84860771820. 22506599 PMC3342519

[24] Bushnell B. B. B. M. , A Fast, Accurate, splice-aware Aligner, 2014, Lawrence Berkeley National Lab.

[25] Gurevich A. , Saveliev V. , Vyahhi N. , and Tesler G. , QUAST: Quality Assessment Tool for Genome Assemblies, Bioinformatics. (2013) 29, no. 8, 1072–1075, 10.1093/bioinformatics/btt086, 2-s2.0-84876266928. 23422339 PMC3624806

[26] Nayfach S. , Camargo A. P. , Schulz F. , Eloe-Fadrosh E. , Roux S. , and Kyrpides N. C. , Checkv: Assessing Viral Genome Quality, Nature Biotechnology. (2021) 39, no. 5, 578–585, 10.1038/s41587-020-00774-7. PMC8116208 33349699

[27] Bouras G. , Nepal R. , Houtak G. , Psaltis A. J. , Wormald P. J. , and Vreugde S. , Pharokka: A Fast Scalable Bacteriophage Annotation Tool, Bioinformatics. (2023) 39, no. 1, 10.1093/bioinformatics/btac776. PMC9805569 36453861

[28] Hunt M. , De Silva N. , Otto T. D. , Parkhill J. , Keane J. A. , and Harris S. R. , Circlator: Automated Circularization of Genome Assemblies, Genome Biology. (2015) 16, no. 1, 10.1186/s13059-015-0849-0, 2-s2.0-84951969455. PMC4699355 26714481

[29] Hockenberry A. J. and Wilke C. O. , BACPHLIP: Predicting Bacteriophage Lifestyle from Conserved Protein Domains, PeerJ. (2021) 9, 10.7717/peerj.11396. PMC8106911 33996289

[30] Shang J. , Jiang J. , and Sun Y. , Phage Classification Using Graph Convolution Networks, Bioinformatics. (2021) 37, no. 1, i25–i33, 10.1093/bioinformatics/btab293. 34252923 PMC8275337

[31] Eddy S. R. , Accelerated Profile HMM Searches, PLoS Computational Biology. (2011) 7, no. 10, 10.1371/journal.pcbi.1002195, 2-s2.0-80055082271. PMC3197634 22039361

[32] Jones P. , Binns D. , Chang H. Y et al., Interproscan 5: Genome-Scale Protein Function Classification, Bioinformatics. (2014) 30, no. 9, 1236–1240, 10.1093/bioinformatics/btu031, 2-s2.0-84899518211. 24451626 PMC3998142

[33] Zankari E. , Hasman H. , Cosentino S et al., Identification of Acquired AMR Genes, Journal of Antimicrobial Chemotherapy. (2012) 67, no. 11, 2640–2644, 10.1093/jac/dks261, 2-s2.0-84867587461. 22782487 PMC3468078

[34] Liu B. , Zheng D. , Jin Q. , Chen L. , and Yang J. , VFDB 2022: A Classification Scheme for Bacterial Virulence Factors, Nucleic Acids Research. (2022) 50, D912–D917. 34850947 10.1093/nar/gkab1107 PMC8728188

[35] Seemann T. , Abricate: Screening Contigs for AMR and Virulence Genes, GitHub. (2022) .

[36] Cook R. , Brown N. , Redgwell T. et al., INPHARED: A Database of Publicly Available Phage Genomes and Metadata, Nucleic Acids Research. (2021) 49, no. D1, D741–D747, 10.1093/nar/gkaa939.

[37] Nishimura Y. , Yoshida T. , Kuronishi M. , Uehara H. , Ogata H. , and Goto S. , Viptree: Viral Proteomic Tree Server, Bioinformatics. (2017) 33, no. 15, 2379–2380, 10.1093/bioinformatics/btx157, 2-s2.0-85026346250. 28379287

[38] Mohapatra R. , Varsani A. , and Kropinski A. M. , VIRIDIC: Calculating Intergenomic Similarities, Viruses. (2020) 12, no. 11. 10.3390/v12111268 PMC7694805 33172115

[39] Page A. J. , Cummins C. A. , Hunt M et al., Roary: Rapid pan-genome Analysis, Bioinformatics. (2015) 31, no. 22, 3691–3693, 10.1093/bioinformatics/btv421, 2-s2.0-84947793096. 26198102 PMC4817141

[40] Hadfield J. , Croucher N. J. , Goater R. J. , Abudahab K. , Aanensen D. M. , and Harris S. R. , Phandango: An Interactive Viewer for Bacterial Population Genomics, Bioinformatics. (2018) 34, no. 2, 292–293, 10.1093/bioinformatics/btx610, 2-s2.0-85040569696. 29028899 PMC5860215

[41] Hackl T. , Martin R. , Ankenbrand M. J. , Hattab G. , and Heider D. , Gggenomes: A Grammar of Graphics for Comparative Genomics, Bioinformatics. (2024) 40, no. 1, 10.1093/bioinformatics/btad767.

[42] Adriaenssens E. M. , Sullivan M. B. , Knezevic P. et al., ICTV Report Consortium. Taxonomy of Prokaryotic Viruses: 2022 Update from the ICTV Bacterial and Archaeal Viruses Subcommittee, Archives of Virology. (2022) 168, no. 2, 10.1007/s00705-022-05694-2.

[43] Strathdee S. A. , Phage Therapy: Mechanisms to Future Directions, Cell Host & Microbe. (2024) . 10.1016/j.cell.2022.11.017 PMC9827498 36608652

[44] Gordillo Altamirano F. L. , Kostoulias X. , Subedi D. et al., Phage-Antibiotic Combination is a Superior Treatment Against *Acinetobacter baumannii* in a Preclinical Study, EBioMedicine. (2024) 103. 10.1016/j.ebiom.2022.104045 PMC9097682 35537278

[45] Forti F. , Roach D. R. , and Cafora M. , Design of a Broad-Range Bacteriophage Cocktail That Reduces *Pseudomonas aeruginosa* Biofilms and Treats Acute Infections in Two Animal Models, Antimicrob Agents Chemother. (2018) 25, no. 6, 10.1128/AAC.02573-17, 2-s2.0-85047635775. PMC5971607 29555626

[46] Labrie S. J. , Samson J. E. , and Moineau S. , Bacteriophage Resistance Mechanisms, Nature Reviews Microbiology. (2010) 8, no. 5, 317–327, 10.1038/nrmicro2315, 2-s2.0-77951104433. 20348932

[47] Gordillo Altamirano F. and Barr J. J. , Phage Therapy in the Post-antibiotic Era, Clinical Microbiology Reviews. (2019) 32, no. 2, e00066–18, 10.1128/cmr.00066-18, 2-s2.0-85060144465. 30651225 PMC6431132

[48] Pires D. P. , Cleto S. , Sillankorva S. , Azeredo J. , and Lu T. K. , Genetically Engineered Phages: A Decade of Advances, Microbiology and Molecular Biology Reviews. (2016) 80, no. 3, 523–543, 10.1128/mmbr.00069-15, 2-s2.0-84991105090. 27250768 PMC4981678

