# Supplementary material for: From Sewage to Salvage: Complete Characterization of Arefeen1 Phage Against MDR Pseudomonas aeruginosa
Source: Int J Microbiol. 2026 Apr 24;2026:8509161. doi: 10.1155/ijm/8509161 (PMC13107164; doi:10.1155/ijm/8509161)
Supplement: Supplementary file 1 — Supporting Information Additional supporting information can be found online in the Supporting Information section. [file IJM-2026-8509161-s001.docx]

|  | 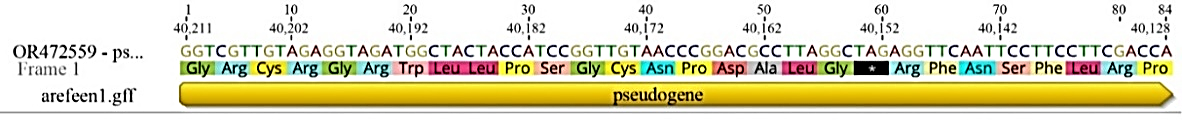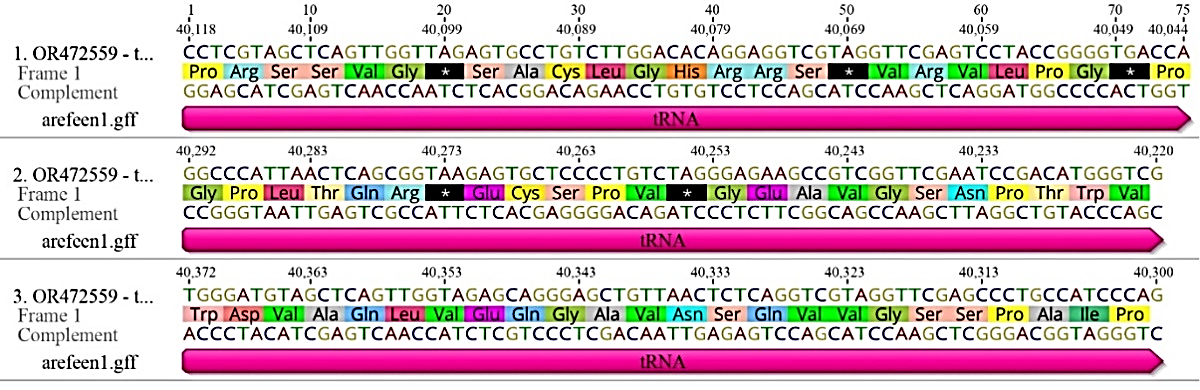 |
| --- | --- |

**Supplimentary Figure 1.** Genome annotation map of phage Arefeen1, indicating the locations of 3 tRNA genes and 1 pseudogene.
